# Caesarean scar pregnancy complicated by partial rupture in the second trimester: A case report

**DOI:** 10.1016/j.crwh.2024.e00665

**Published:** 2024-11-12

**Authors:** Prakriti Garkhail, Astrid S.M. Vinkesteijn, Sabina de Weerd

**Affiliations:** a Department of Obstetrics and Gynaecology, Albert Schweitzer hospital, Albert Schweitzerplaats 25, 3318 AT Dordrecht, the Netherlands; b Department of Gynaecologic Oncology, Erasmus MC Cancer Institute, University Medical Centre Rotterdam, Dr. Molewaterplein 40, 3015 GD Rotterdam, the Netherlands

**Keywords:** Caesarean, Scar, Pregnancy, Niche, Uterine, Rupture

## Abstract

This case report examines caesarean scar pregnancy, a rare but significant complication associated with increasing global caesarean rates. It explores diagnostic challenges, therapeutic interventions, and the importance of a multidisciplinary approach. This report details the case of a patient at 13 + 4 weeks of amenorrhea presenting with severe abdominal pain, diagnosed with caesarean scar pregnancy and scar dehiscence causing major haemorrhage. Emergency surgery and interventional radiology were employed for pregnancy evacuation and haemorrhage control. This report emphasizes niche pregnancy complexities and underscores the need for evidence-based practices to mitigate maternal morbidity and mortality. Additionally, it emphasizes the need for training in niche detection by transvaginal ultrasound for all clinicians encountering patients with early pregnancies.

## Highlights

• Caesarean scar pregnancies pose challenges and require specialist teamwork.
• Interventional radiology plays a key role in managing this complex obstetric issue.
• Routine niche detection techniques are key for clinicians treating pregnant patients.
• Combined efforts to reduce caesarean sections can lower the risk of caesarean scar pregnancy.

## 1 Introduction

Caesarean scar pregnancy (CSP) is a rare complication that has potential for catastrophic outcomes. CSP occurs when a fertilized egg implants within or adjacent to the scar tissue from a previous caesarean section (CS), resulting in a pregnancy that develops within the myometrium at the uterine scar [1]. Due to rising caesarean deliveries worldwide [2], late complications such as CSP have also increased, with an incidence of one in every 531 pregnancies after CS [3,4]. A possible outcome of CSP is uterine rupture, which can lead to profound maternal morbidity, emergency hysterectomy and mortality [5]. CSP's variable clinical features make diagnosis and treatment challenging [6]. Thus far, there is no universal guideline for CSP diagnosis and treatment [5]. Current treatment usually consists of terminating the pregnancy through methotrexate injections or transvaginal or laparoscopic resection [3,4,7]. The objective of this case report is to contribute to the growing body of knowledge regarding the presentation, diagnosis, and management of CSP with a focus on identifying and treating uterine rupture in this context.

## 2 Case Presentation

A patient in her 30s, gravida 4 para 2, amenorrhea 13 + 4 weeks, was admitted to the emergency unit by ambulance with severe abdominal pain and tachycardia. She had a history of a third-degree perineal tear during her first vaginal delivery and a subsequent CS during her second pregnancy, with no history of comorbidities. Four months prior to presentation, she had undergone an abortion by dilatation and curettage (D&C). One week prior to presentation, she had been seen by her midwife for a routine check-up, where no abnormalities had been noted.

At presentation, a FAST ultrasound (Focused Assessment with Sonography in Trauma) revealed free fluid in the abdomen extending up to the liver edges, and a significant drop in haemoglobin levels was noted compared with one week earlier, from 7.3 mmol/L (11.8 g/dL) to 6.3 mmol/L (10.6 g/dL). Initial management of this acute bleeding included intravenous fluids and a transfusion of O-negative packed red blood cells, after which a further drop in haemoglobin to 5.9 mmol/L (9.5 g/dL) was measured. The patient was showing signs of hypovolemic shock, presenting with tachypnea under oxygen at a flow rate of 12 L per minute via a non-rebreathing mask, normal blood pressure and tachycardia at 110 beats per minute (bpm), amounting to a National Early Warning Score (NEWS) 2 of 5 [8].

**Fig. 1:**
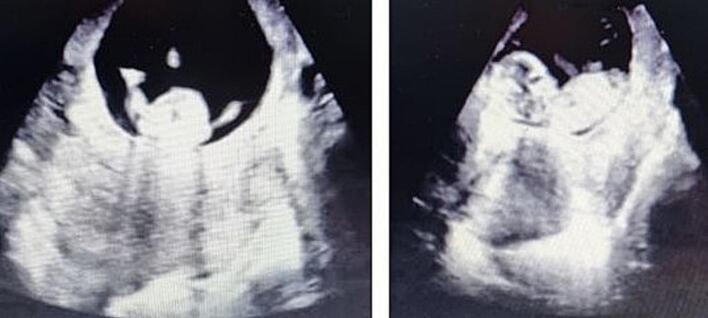
Abdominal ultrasound image of intact pregnancy with thin anterior uterine wall.

Upon further emergency evaluation by the gynaecology team, transvaginal ultrasound revealed free fluid pooling in the rectouterine pouch. Abdominal ultrasound showed a normal foetal heart rate of 145 bpm, no evident signs of uterine rupture and a thin anterior uterine wall (Fig. 1). The on-call gynaecologist, in collaboration with general surgeons, initiated diagnostic laparoscopy, during which approximately two litres of blood was drained from the abdominal cavity. A uterine bleed was identified, and attempts to control the bleeding using a gelatine-based haemostatic agent proved insufficient due to suboptimal visualization. Consequently, the decision was made to convert to a laparotomy through a medial incision.

### 2.1 Differential Diagnosis

The differential diagnosis at the time was a CSP, a spontaneous haemoperitoneum in pregnancy (SHiP) or, less likely, a non-traumatic bleed from other abdominal organs, such as the liver or spleen. A SHiP is usually a non-traumatic bleed during pregnancy and is associated with endometriosis [9]. The patient did not have a history of endometriosis. Due to the life-threatening nature of the situation, an extensive differential diagnosis was not considered and the clinical team opted to perform emergency surgery to visualize the source of the bleed.

### 2.2 Treatment and Outcomes

**Fig. 2:**
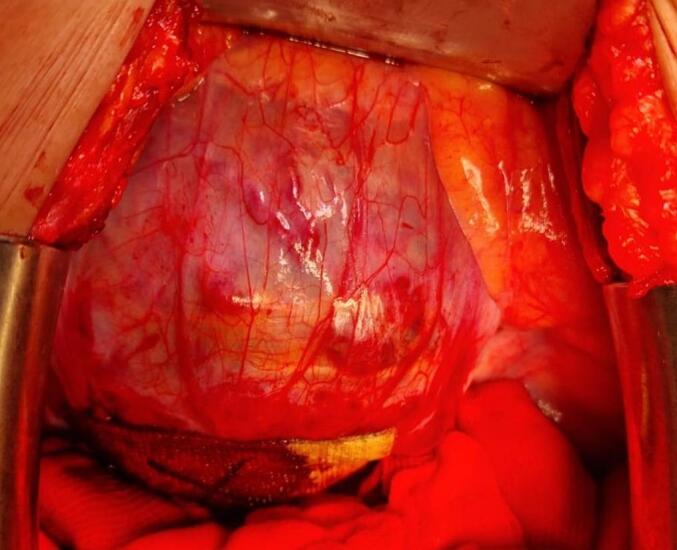
Foetal membranes protruding through the dehiscence during laparotomy.

During the laparotomy, a CSP was discovered within the uterine scar of the previous caesarean section, resulting in scar dehiscence. The foetal membranes were observed protruding through the dehiscence, with only a thin layer of serosa covering them *(*Fig. 2*)*. Damage in this serosal layer, most probably due to the rapidly growing, gravid uterus, led to multiple small bleeding lesions. Video-consultation with a nearby academic centre led to a joint decision to attempt evacuation of the pregnancy, accepting the risk of the necessity of a hysterectomy in case of major haemorrhage. To ultimately reduce the risk of expected heavy bleeding, an interventional radiologist was called in to place sheaths and occluding balloons in the internal iliac arteries. This took place in the operation room, after which the procedure could be continued. During this time, the patient remained stable.

**Fig. 3:**
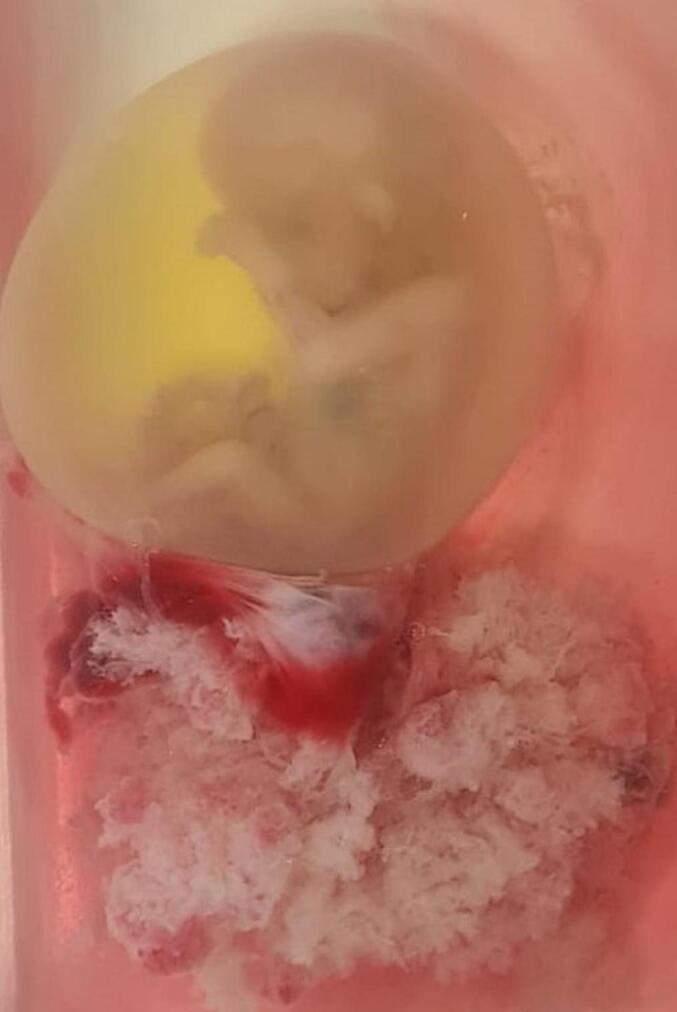
Foetus amenorrhea 13 + 4 evacuated intact within membranes.

Using blunt dissection, an approximately 3 cm opening was created in the serosal layer covering the pregnancy, allowing for the successful delivery of the pregnancy intact within the membranes *(*Fig. 3*)*. As anticipated, post-placental removal, the lower segment of the uterus continued to bleed. To control the haemorrhage, the uterus was closed in one layer and a Foley catheter was inserted vaginally and inflated, applying pressure internally to the uterus. A gauze compress was placed intravaginally, and the patient was subsequently transferred to the intensive care unit (ICU). Estimated total blood loss during the procedure was three litres, for which the patient was transfused and treated with tranexamenic acid and calcium.

In the ICU the patient remained stable and the arterial sheaths and vaginal catheter were removed the following day, after which she was successfully extubated. The patient did not experience any complications due to this procedure and could be discharged from the hospital after 6 days. At an outpatient follow-up visit four weeks postoperatively she reported no physical complications. She was advised against future pregnancy and reported no residual complaints six months postoperatively.

## 3 Discussion

### 3.1 Diagnostic Challenges

CSP is an abnormally located pregnancy in the lower uterine cavity. In a normal pregnancy, penetration of the trophoblast into the uterine wall is stopped by a thin mucosal layer: the decidua basalis. Cases of CSP are usually preceded by a placenta accreta or percreta, which is defined by abnormal growth of the placenta into the uterus [10]. It occurs at a cellular level due to the decidua basalis being extremely thin or absent which can occur due to surgery and scarring [11]. This can cause the pregnancy to grow into the myometrium and nestle into or on the scar of a previous CS.

Accurate and timely diagnosis of CSP is crucial. Patients often present with vaginal bleeding or pain in the first trimester [10]. Transvaginal ultrasound (TVUS) is the primary imaging tool for early detection and has proven to be reliable [12]. Magnetic resonance imaging (MRI) could be beneficial in non-emergency situations when ultrasound remains inconclusive [13]. Awareness of CSP detection is essential. The patient in the reported case underwent multiple first-trimester scans at her midwifery practice with no abnormal findings. A recent modified Delphi study showed that experts believe basic ultrasound to detect CSP should always be conducted [14]. This is an elaborate process and needs extensive training. It is important that all clinicians encountering patients early in pregnancy, including at midwifery practices and ultrasound clinics, have awareness of the diagnostic criteria for CSP and be trained in seeking for niche in patients with a history of CS.

### 3.2 Therapeutic Strategies

CSP treatment often consists of terminating the pregnancy through intramuscular and intragestational methotrexate injection, transvaginal resection, laparoscopy or uterine artery embolization with D&C and hysteroscopy [3,4,7]. In this case, laparoscopy was initiated as a less invasive method, but conversion to laparotomy was necessary due to suboptimal visualization. There seems to be a preference for an interventional approach versus an expectant approach. However, the level of evidence is low, consisting of mainly case series [7]. Multicentre studies are needed to formulate evidence-based treatment guidelines.

### 3.3 Management of Haemorrhage

Massive haemorrhage is believed to account for a quarter of annual maternal deaths and 8 % of maternal deaths in The Netherlands [15,16]. Therefore it is essential to keep searching for optimal techniques to improve the management of haemorrhage, in spite of gestational age and cause. Interventional radiology has become increasingly important in managing obstetric emergencies, including CSP. Pelvic arterial embolization, first reported in 1979 [17], plays a crucial role in managing haemorrhage in obstetric care. In 2007 the Royal College of Obstetricians and Gynaecologists published a guideline advising the pre-emptive use of intervention radiology in the management of obstetric cases where postpartum haemorrhage is likely [18]. In the present case, interventional radiology was consulted for pre-emptive arterial sheath placement.

Balloon occlusion was used in the iliac arteries to prevent excessive haemorrhage. The use of balloon-occlusion by catheter for obstetric haemorrhage has been applied to reduce blood loss during surgery in the past two decades [19,20]. The most important advantages are fast on-site placement and reversibility post-surgery. Complications are rare but can be severe, such as acute limb ischemia due to thromboembolism and aneurysm [21]; the supporting evidence consists mainly of small cohort studies with varying results [20,22]. Further, prospective analysis to evaluate the risk-benefit ratio is necessary.

After evacuation of the pregnancy in the present case, a Foley balloon catheter was placed in utero to tamponade the bleed. This was effective and showed no adverse effects. In light of this successful experience, as well as supporting literature [23], the advice would be to continue using this method during the management of first- and early second-trimester CSP.

### 3.4 Reducing Caesarean Sections

According to the World Health Organization, in 2021 about 21 % of deliveries worldwide were done by CS, whereas in 1990 this was about 7 % [2]. The rising global trend has prompted discussions on the importance of reducing unnecessary CS. While sometimes medically necessary, CS carries inherent risks, including potential complications in subsequent pregnancies, such as CSP. Multifaceted strategies with audit and detailed feedback seem to be the most effective to reduce CS rates, and identifying barriers to change is an important first step in the process (24). These efforts can contribute to a decreased incidence of niche pregnancies and their associated risks.

## Appendix A Supplementary data

Supplementary material Image 1

## References

[1] Lai Y.M., Lee J.D., Lee C.L., Chen T.C., Soong Y.K. (1995). An ectopic pregnancy embedded in the myometrium of a previous cesarean section scar. Acta Obstet. Gynecol. Scand..

[2] Organization WH (2021). Caesarean Section Rates Continue to Rise, amid Growing Inequalities in Access. https://www.who.int/news/item/16-06-2021-caesarean-section-rates-continue-to-rise-amid-growing-inequalities-in-access.

[3] Timor-Tritsch I.E., Monteagudo A., Santos R., Tsymbal T., Pineda G., Arslan A.A. (2012). The diagnosis, treatment, and follow-up of cesarean scar pregnancy. Am. J. Obstet. Gynecol..

[4] Bodur S., Ozdamar O., Kilic S., Gun I. (2015). The efficacy of the systemic methotrexate treatment in caesarean scar ectopic pregnancy: a quantitative review of English literature. J. Obstet. Gynaecol..

[5] Dior U.P., Palma-Dias R., Reidy K.L., Cheng C., Healey M. (2019). Cesarean scar pregnancies: incidence and factors associated with conversion to surgery from medical management. J. Minim. Invasive Gynecol..

[6] Granese R., Gulino F.A., Incognito G.G., Martinelli C., Cianci S., Ercoli A. (2023). Scar pregnancy: a rare, but challenging, obstetric condition. J. Clin. Med..

[7] Birch Petersen K., Hoffmann E., Rifbjerg Larsen C., Svarre Nielsen H. (2016). Cesarean scar pregnancy: a systematic review of treatment studies. Fertil. Steril..

[8] Smith G.B., Redfern O.C., Pimentel M.A., Gerry S., Collins G.S., Malycha J., Prytherch D., Schmidt P.E., Watkinson P.J. (2019). The National Early Warning Score 2 (NEWS2). Clin. Med. (Lond.).

[9] Lier M.C.I., Malik R.F., Ket J.C.F., Lambalk C.B., Brosens I.A., Mijatovic V. (2017). Spontaneous hemoperitoneum in pregnancy (SHiP) and endometriosis - a systematic review of the recent literature. Eur. J. Obstet. Gynecol. Reprod. Biol..

[10] Horgan R., Abuhamad A. (2022). Placenta accreta spectrum: prenatal diagnosis and management. Obstet. Gynecol. Clin. N. Am..

[11] Timor-Tritsch I.E., Monteagudo A., Cali G., D’Antonio F., Kaelin Agten A. (2019). Cesarean scar pregnancy: diagnosis and pathogenesis. Obstet. Gynecol. Clin. N. Am..

[12] Liu D., Yang M., Wu Q. (2018). Application of ultrasonography in the diagnosis and treatment of cesarean scar pregnancy. Clin. Chim. Acta.

[13] Osborn D.A., Williams T.R., Craig B.M. (2012). Cesarean scar pregnancy: sonographic and magnetic resonance imaging findings, complications, and treatment. J. Ultrasound Med..

[14] Jordans I.P.M., Verberkt C., De Leeuw R.A., Bilardo C.M., Van Den Bosch T., Bourne T. (2022). Definition and sonographic reporting system for cesarean scar pregnancy in early gestation: modified Delphi method. Ultrasound Obstet. Gynecol..

[15] Khan K.S., Wojdyla D., Say L., Gulmezoglu A.M., Van Look P.F. (2006). WHO analysis of causes of maternal death: a systematic review. Lancet.

[16] Kallianidis A.F., Schutte J.M., Schuringa L.E.M., Beenakkers I.C.M., Bloemenkamp K.W.M., Braams-Lisman B.A.M., Cornette J., Kuppens S.M., Rietveld A.L., Schaap T., Stekelenburg J., Zwart J.J., van den Akker T. (2022 Apr). Confidential enquiry into maternal deaths in the Netherlands, 2006-2018. Acta Obstet. Gynecol. Scand..

[17] Heaston D.K., Mineau D.E., Brown B.J., Miller F.J. (1979). Transcatheter arterial embolization for control of persistent massive puerperal hemorrhage after bilateral surgical hypogastric artery ligation. AJR Am. J. Roentgenol..

[18] Gynaecologists RCoOa (2007). Royal College of Obstetricians and Gynaecologists Good Practice Guideline No 6.

[19] Dubois J., Garel L., Grignon A., Lemay M., Leduc L. (1997). Placenta percreta: balloon occlusion and embolization of the internal iliac arteries to reduce intraoperative blood losses. Am. J. Obstet. Gynecol..

[20] Levine A.B., Kuhlman K., Bonn J. (1999). Placenta accreta: comparison of cases managed with and without pelvic artery balloon catheters. J. Matern. Fetal Med..

[21] Sewell M.F., Rosenblum D., Ehrenberg H. (2006). Arterial embolus during common iliac balloon catheterization at cesarean hysterectomy. Obstet. Gynecol..

[22] Bodner L.J., Nosher J.L., Gribbin C., Siegel R.L., Beale S., Scorza W. (2006). Balloon-assisted occlusion of the internal iliac arteries in patients with placenta accreta/percreta. Cardiovasc. Intervent. Radiol..

[23] Timor-Tritsch I.E., Cali G., Monteagudo A., Khatib N., Berg R.E., Forlani F., Avizova E. (2015). Foley balloon catheter to prevent or manage bleeding during treatment for cervical and cesarean scar pregnancy. Ultrasound Obstet. Gynecol..

