# Supplementary material for: Caesarean scar pregnancy complicated by partial rupture in the second trimester: A case report
Source: Case Rep Womens Health. 2024 Nov 12;44:e00665. doi: 10.1016/j.crwh.2024.e00665 (PMC11609501; doi:10.1016/j.crwh.2024.e00665)
Supplement: Supplementary file 1 — Supplementary material [file mmc1.docx]

**Patient perspective**

Written by the patient, translated and edited by author PG:

My husband and I had been very scared about this pregnancy. A lot of times there was some blood loss and we went for check-ups to our midwife. There everything looked perfect. It was a difficult time for us, as my father had died as well, but this baby was a gift we got back. At the 13-week ultrasound we found out that it was to be a little boy, who we would name after my father. The next day my life changed, I thought I was ill due to something I ate, but I nearly died and my husband and children nearly lost their wife and mother. It has been an ordeal on me and my family to recover from this. I was bedridden for 4-6 weeks, my husband could not work and I still have nightmares of the intensive care. I am thankful to the hospital for saving my life, but I want to stress the importance of diagnosing these cases in midwifery practices as well!
